# Generation of Human Melanocytes from Induced Pluripotent Stem Cells

**DOI:** 10.1371/journal.pone.0016182

**Published:** 2011-01-13

**Authors:** Shigeki Ohta, Yoichi Imaizumi, Yohei Okada, Wado Akamatsu, Reiko Kuwahara, Manabu Ohyama, Masayuki Amagai, Yumi Matsuzaki, Shinya Yamanaka, Hideyuki Okano, Yutaka Kawakami

**Affiliations:** 1 Division of Cellular Signaling, Institute for Advanced Medical Research, Keio University School of Medicine, Tokyo, Japan; 2 Department of Physiology, Keio University School of Medicine, Tokyo, Japan; 3 Kanrinmaru Project, Keio University School of Medicine, Tokyo, Japan; 4 Department of Dermatology, Keio University School of Medicine, Tokyo, Japan; 5 Center for iPS Cell Research and Application (CiRA), Kyoto University, Kyoto, Japan; 6 Institute for Integrated Cell-Material Sciences, Kyoto University, Kyoto, Japan; Genome Institute of Singapore, Singapore

## Abstract

Epidermal melanocytes play an important role in protecting the skin from UV rays, and their functional impairment results in pigment disorders. Additionally, melanomas are considered to arise from mutations that accumulate in melanocyte stem cells. The mechanisms underlying melanocyte differentiation and the defining characteristics of melanocyte stem cells in humans are, however, largely unknown. In the present study, we set out to generate melanocytes from human iPS cells *in vitro*, leading to a preliminary investigation of the mechanisms of human melanocyte differentiation. We generated iPS cell lines from human dermal fibroblasts using the Yamanaka factors (SOX2, OCT3/4, and KLF4, with or without c-MYC). These iPS cell lines were subsequently used to form embryoid bodies (EBs) and then differentiated into melanocytes via culture supplementation with Wnt3a, SCF, and ET-3. Seven weeks after inducing differentiation, pigmented cells expressing melanocyte markers such as MITF, tyrosinase, SILV, and TYRP1, were detected. Melanosomes were identified in these pigmented cells by electron microscopy, and global gene expression profiling of the pigmented cells showed a high similarity to that of human primary foreskin-derived melanocytes, suggesting the successful generation of melanocytes from iPS cells. This *in vitro* differentiation system should prove useful for understanding human melanocyte biology and revealing the mechanism of various pigment cell disorders, including melanoma.

## Introduction

Pigmented cells including epidermal melanocytes play an important physiological role in providing protection from harmful ultraviolet rays, and are also implicated in various pigmented cell disorders. Either defects in, or a lack of melanocytes and/or melanocyte stem cells (MELSCs) can lead to pigment disorders such as piebaldism, albinism, vitiligo, and hair graying. Vitiligo is a common disease affecting approximately 0.1 to 2.0% of the world population, although the pathogenesis has not completely understood [1]. Autologous cultured melanocytes may be useful for the treatment of vitiligo [2], [3]. In contrast to foreskin melanocytes, expansion of adult melanocytes is quite difficult. Thus, development of methods to generate large numbers of autologous melanocytes is required. Among pigment cell disorders, melanoma is one of the most aggressive types of human cancers, and is suspected to arise from MELSCs. The recent progress of cancer stem cell studies supports the hypothesis that melanoma stem cells (MMSCs) which are resistant to chemotherapy may exist, and are thus important therapeutic targets [4], [5]. It is proposed that MMSCs are generated from MELSCs through accumulation of genetic changes and may have similar phenotypes to MELSCs [6]. Thus, understanding the biology of human MELSCs and MMSCs is critically important. In addition, to permit the investigation of MELSCs and MMSCs, their purification or generation is required. To date, some candidate markers for MELSCs (DCT; dopachrome tautomerase, and PAX3) [7], [8] and MMSCs (ABCB5, CD20, CD133, CD271) [9], [10], [11], [12] have been reported, respectively; however, their specificity is still controversial. Refining such knowledge will permit the development of therapeutic treatments against MMSCs, including immunotherapy.

Melanocytes are specialized cells derived from the neural crest cells during embryonic development that migrated to hair follicles and basal layer of the epidermis. A number of studies have shown that cell factors such as MITF, c-Kit, and Snail/Slug are important for melanocyte development. Especially, MITF can regulate the melanocyte lineage in part by regulating several pigmentation enzymes including DCT, TYRP1, and tyrosinase [13]. It has been reported that MELSCs locate in the bulge region of hair follicles in mice [7], however, the localization of MELSCs in human skin has been unclear due to lack of definitive markers. It is also difficult to analyze the developmental cell lineages of skin melanocytes in humans. Thus, it is desirable to develop a new *in vitro* system for generating human melanocytes through MELSCs, which mimics *in vivo* differentiation processes to better understand human melanocyte development. Melanocyte generation from embryonic stem (ES) cells has been previously reported [14], [15]. Alternatively, induced pluripotent stem (iPS) cells have specific advantages compared to ES cells. Besides avoiding ethical issues, iPS cells can be propagated as autologous cells, meaning that melanocytes from autologous iPS cells are not likely to be immunologically rejected if transplanted for the treatment pigment cell disorders. In addition, melanocytes may be generated from the iPS cells of patients with genetic pigment cell disorders, leading the understanding of mechanisms of the diseases, as shown previously for ALS, Familial dysautonomia, Parkinson's disease and SMA [16], [17], [18], [19]. With these considerations, human iPS cells are a superior starting cell source to generate melanocytes through neural crest cells *in vitro*, leading to a better understanding of the characteristics of MELSCs and MMSCs.

In this study, we have established an *in vitro* system for generating human melanocytes from iPS cells, apparently through a neural crest cell intermediate. This system may contribute to the understanding of human melanocyte development and various pigment cell disorders, including melanoma. It may also be useful for the preparation of large numbers of autologous melanocytes for treating hypopigmental diseases.

## Results

### Generation of human iPS cells from human dermal fibroblasts

**Figure 1:**
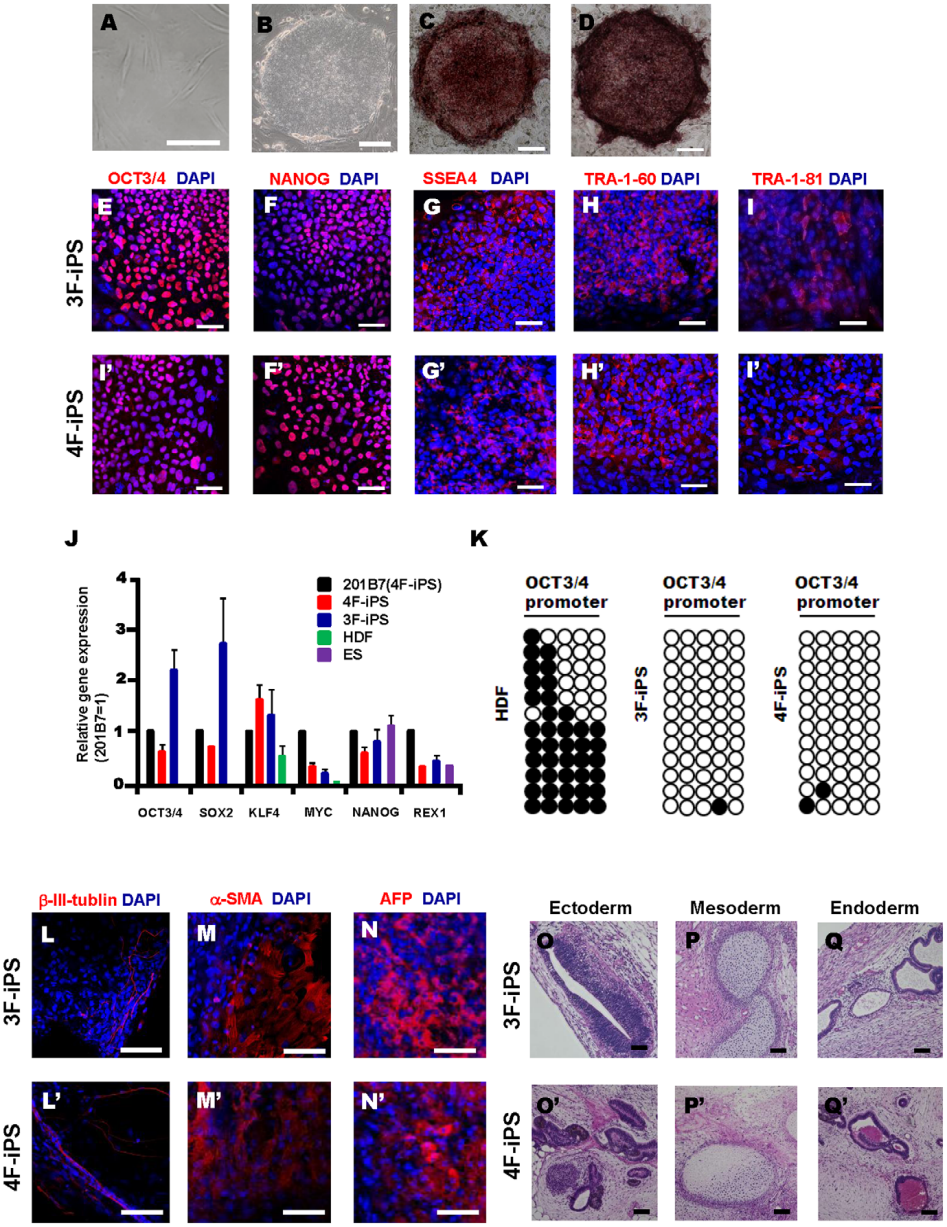
Induction of human 3F- and 4F-iPS cells from adult dermal fibroblasts and characterization of their pluripotency. (A) Morphology of human dermal fibroblasts. (B) Representative image of an established iPS cell colony cultured on mitomycin C-treated STO feeders. (C, D) Alkaline phosphatase (AP) staining in a 3F- (C) and 4F- (D) iPS cell colony. (E–I′) Expression of pluripotency markers in established 3F- and 4F-iPS colonies. 3F-iPS (E–I) and 4F-iPS (E′–I′) colonies express markers common to pluripotent cells including OCT3/4, NANOG, SSEA-4, TRA-1-60, and TRA-1-81. DAPI (4, 6-diamidino-2-phenylindole) staining indicates the total cell content per field. Scale bars, 20 µm (A), 100 µm (B, C, and D), 50 µm (E–I′). (J) Expression of pluripotency genes. Endogenous gene expression levels of OCT3/4, SOX2, KLF4, c-MYC, NANOG, and REX1 were determined by quantitative PCR in parental human dermal fibroblasts (HDF), 3F- and 4F-iPS cells, 201B7 (4F-iPS cells) [20] and ES cells (KhES-1). The graphs show the average of two independent experiments. Error bar indicates mean±S.E.M. (K) iPS cells are demethylated at the OCT3/4 promoter relative to their parental fibroblasts. Bisulfite sequencing analysis of the OCT3/4 promoter in parental HDFs, 3F-, and 4F-iPS cells. Each horizontal row of circles represents an individual sequencing reaction for a given amplification. White circles represent unmethylated CpG dinucleotides; black circles represent methylated CpG dinucleotides. (L–N′) Images of differentiated cells at day 12. 3F- (L–N) and 4F-iPS (L′–N′) cells were cultured in suspension to form EBs for three weeks and then transferred to gelatin-coated plates and cultivated for another 12 days. Immunocytochemical analyses showed positive cells in spontaneously differentiated iPS cell colonies for β-III tubulin (ectoderm, L and L′), α-smooth muscle actin (α-SMA, mesoderm, M and M′), and α-fetoprotein (AFP, endoderm, N and N′). Scale bar, 50 µm. (O–Q′) Generation of teratoma-like masses in testis by xenograft of 3F- and 4F-iPS. Paraffin-embedded sections were stained with hematoxylin and eosin. Resulting teratomas display features of ectoderm (O and O′), mesoderm (P and P′) and endoderm (Q and Q′). Scale bar, 100 µm.

We established two human iPS cell lines following the methods established by Takahashi *et al.* [20] from dermal fibroblasts using all four retrovirally expressed Yamanaka factors (4F) (SOX2, OCT3/4, KLF4, c-MYC) or using three factors (3F) without c-MYC (Figure 1A and 1B). To examine the expression of pluripotency markers in both 3F-iPS cells and 4F-iPS cells, we performed alkaline phosphatase staining (Figure 1C and 1D) and immunocytochemical analyses on OCT3/4, NANOG, SSEA4, TRA-1-60, and TRA-1-81 in 3F and 4F-iPS cell colonies (Figure 1E–I′). In addition, the gene expression profiles of pluripotent stem cell markers including the endogenous Yamanaka factors, NANOG and REX1 were analyzed in 3F and 4F-iPS cells by quantitative PCR analyses and compared to 4F-iPS cells' parental human dermal fibroblasts and previously characterized 4F-iPS cells, 201B7 [20]. The generated 3F- and 4F-iPS cells expressed endogenous pluripotency markers similarly to 201B7 iPS cells and ES cells (Figure 1J). Transgene expression was also examined using quantitative PCR analyses, indicating a similarly low gene expression level in the both established 3F- and 4F-iPS cells as compared to 201B7 iPS cells, apart from a higher expression of retroviral KLF4 in 3F-iPS cells (Supplemental Figure S1). Evaluation of the methylation status of cytosine guanine dinucleotides (CpG) in the promoter regions of OCT3/4 was analyzed by bisulfite sequencing and compared to 4F-iPS parental dermal fibroblasts. 3F- and 4F-iPS cells were highly unmethylated in comparison (Figure 1K). Furthermore, the differentiation ability of both 3F- and 4F-iPS cells was examined *in vitro*. Floating embryoid bodies (EBs) were cultivated for 3 weeks from each iPS cell set, and then spontaneously differentiated onto gelatin coated dishes for 12 days. Attached cells were immunopositive for three germ cell layers' markers (β-III-tubulin, ectoderm; α-smooth muscle actin, mesoderm; α-fetoprotein, endoderm) (Figure 1L-1N′). Finally, to confirm the pluripotency of 4F-iPS cells *in vivo*, we transplanted 3F- and 4F-iPS cells into the testis of immunodeficient mice (NOD-SCID) to observe teratoma formation. Tumors formed eight weeks after injection and contained various tissues representing all three germ layers (ectoderm, mesoderm, and endoderm; Figure 1O–1Q′) and various tissues (Supplemental Figure S2). Collectively, these results confirmed the establishment of de novo iPS cells from human dermal fibroblasts.

### Melanocyte differentiation from human iPS cells

**Figure 2:**
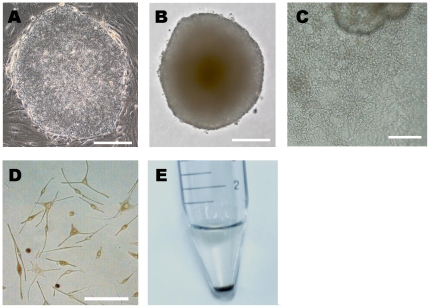
Generation of melanocytes from iPS cells. Efficient differentiation of human iPS cells into pigmented melanocytes in defined conditions. (A) A representative image of a 3F-iPS cell colony grown on a Mitomycin-C-treated STO feeder layer. (B) Embryoid bodies (EBs) formed in suspension under feeder-free conditions. (C) After one week in differentiation medium, cells were observed migrating out from EBs on a fibronectin–coated plate. (D) Morphology of putative melanocytes differentiated from human EBs 7 weeks after differentiation. Cells display melanocytic morphology and pigmentation. (E) Pigmented cell pellet of iPS cell-derived melanocytes 9 weeks after differentiation. Scale bar, 100 µm (A, B, C), 40 µm (D).

Human 3F- and 4F-iPS cells were cultured as colonies on mitomycin-C treated feeder cells in iPS medium containing FGF-2 (Figure 2A). To generate melanocytes, EBs were generated from iPS cells grown in suspension in bacterial culture dishes without FGF-2 for 3 weeks (Figure 2B) and then plated onto fibronectin-coated dishes in melanocyte differentiation medium containing Wnt3a, SCF, ET3, FGF-2, and cAMP inducers (cholera toxin) as described in Materials and Methods. A week after plating, many cells migrating out from EBs were observed (Figure 2C), and culture was continued for an additional 3 weeks, changing the medium every 2–3 days. After this period, whole attached cells were dissociated into single cells using TrypLE Select and replated onto a fibronectin-coated dish in the same melanocyte differentiation medium. Proliferating cells were passaged before reaching confluence. Pigmented cells usually appeared 3–4 weeks after single cell dissociation, and most of the cells were highly pigmented 4–6 weeks after single cell dissociation (Figure 2D, 2E). Using our established system, we can produce approximately 5×10^6^ melanocytes cells based on the percentage of SILV^+^ cells in the total cell number three months after differentiation from a 10 cm cell dish containing confluent iPS cell colonies (approximately 2–3×10^6^ starting iPS cells). We could usually maintain the culture for a few months. However, proliferative capacity of the cells was eventually lost, as is typically seen in primary human melanocyte culture.

### Characterization of human melanocytes derived from iPS cells

**Figure 3:**
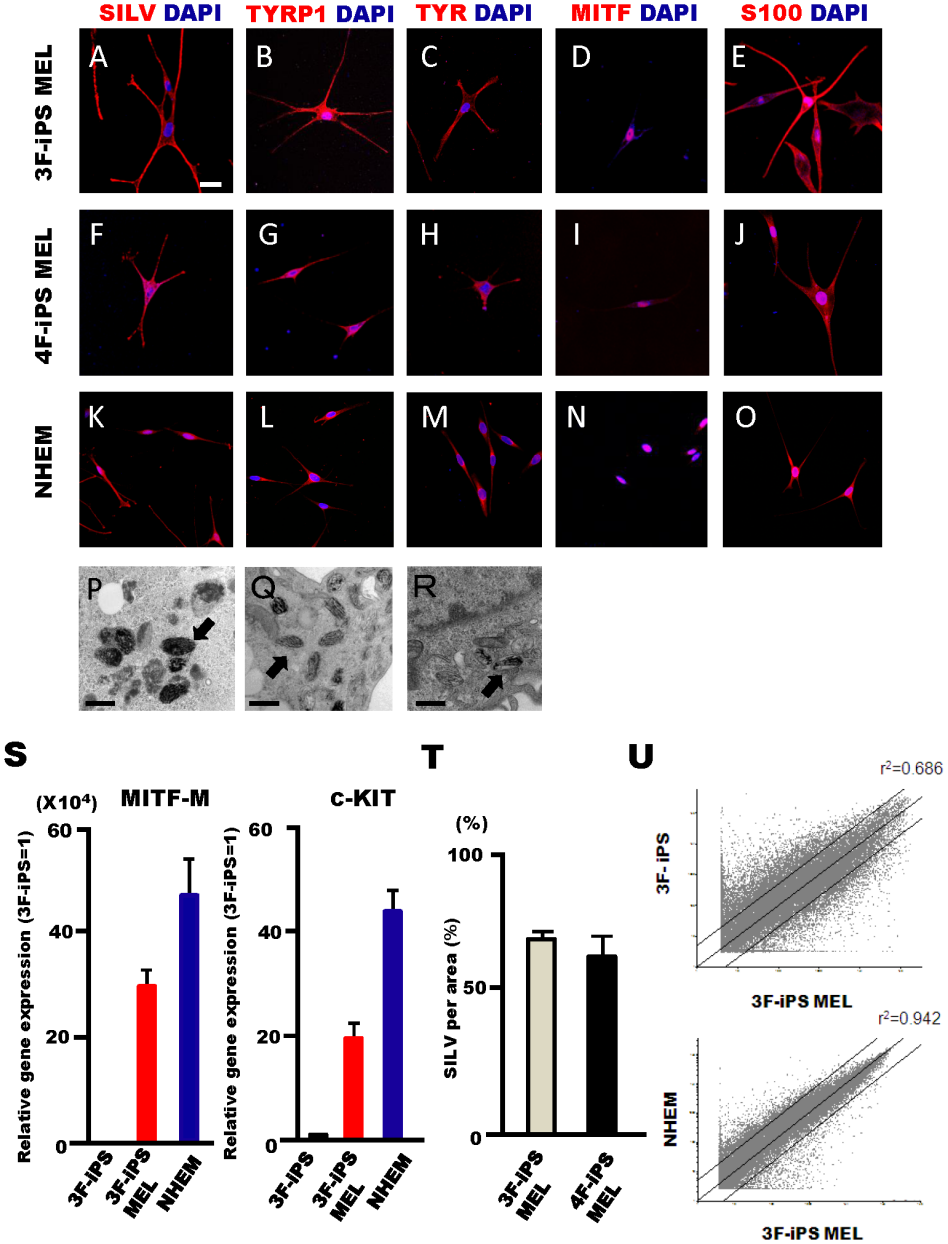
Marker expression characteristic of the melanocyte lineage in human iPS cell-derived melanocytes observed 8 weeks after differentiation. Either 3F- (3F-iPS MEL, A–E) or 4F- (4F-iPS MEL, F–J) iPS cells derived melanocytes were positive for an array of melanocyte markers: SILV (silver protein), TYRP1 (tyrosinase-related protein-1), TYR (tyrosinase), MITF (microphthalmia-associated transcription factor) , and S100. Normal human epidermal melanocytes (NHEM) were also stained with the same antibodies (K–O). Transmission electron microscopy images of either 3F (P), 4F (Q)-iPS cells derived melanocytes, or NHEM (R) showed that those melanocytes have many melanosomes in the cytoplasm. Scale bar, 20 µm (A–O), 0.5 µm (P, Q, and R). (S) Quantitative real-time PCR analysis of MITF-M and c-Kit in 3F-iPS, 3F-iPS MEL, and NHEM. The data are normalized to GAPDH and represented as fold change relative to RNA levels in 3F-iPS. The graphs show the average of two independent experiments. Error bar indicates mean±S.E.M. (T) Quantitation of SILV-positive cells induced from 3F and 4F-iPS cells. Percentage of SILV positive cells upon total differentiation of EBs cells 7 weeks after differentiation under melanocyte differentiation medium. Error bar indicates mean±S.E.M (3F-iPS, n = 3; 4F-iPS, n = 2). (U) The global gene-expression patterns were compared between human 3F-iPS cells and 3F-iPS derived-MEL (3F-iPS MEL), and between NHEM and 3F-iPS MEL. The lines indicate the linear equivalent and 5-fold differences on either side in gene expression levels between the two samples.

To characterize putative melanocytes derived from both 3F- and 4F-iPS cells, we performed immunocytochemical analyses using established melanocyte markers. Each set of iPS cell-derived melanocytes was immune-positive for SLIV, TYRP1, TYR, MITF and S100 antibodies 8 weeks after differentiation (Figure 3A–J). As a positive control, we analyzed normal human foreskin-derived epidermal melanocyte (NHEM) cells isolated from newborn foreskin using the same antibodies (Figure 3K–O). To further confirm the generation of melanocytes, melanosome formation in the pigmented cells was evaluated using transmission electron microscopy, revealing melanosomes in both 3F- and 4F-iPS-derived melanocytes (Figure 3P and 3Q) as seen in NHEM (Figure 3R). In addition, transcripts of MITF-M, which is a MITF gene-isoform known to be expressed in melanocytes and c-Kit that is a marker of melanoblasts and melanocytes were expressed in 3F-iPS cell derived melanocyes (Figure 3S). To compare the efficiency of melanocyte generation from 3F- and 4F-iPS cells, the population of SILV-positive cells was compared 7 weeks after cell differentiation and the almost same differentiation efficiency was observed between 3F- and 4F-iPS derived cells (Figure 3T). Finally, scatter plot analyses were performed using DNA microarray to compare the global gene expression between NHEM and melanocytes derived from 3F-iPS cells at 8 weeks after differentiation, and a tight correlation in the gene expression profile was observed between those samples. These data strongly support that our differentiation conditions are capable of directing the generation of NHEM-like epidermal melanocytes from iPS cells (Fig. 3U).

### iPS cells differentiate into cells expressing neural crest and melanocyte stem cell markers

**Figure 4:**
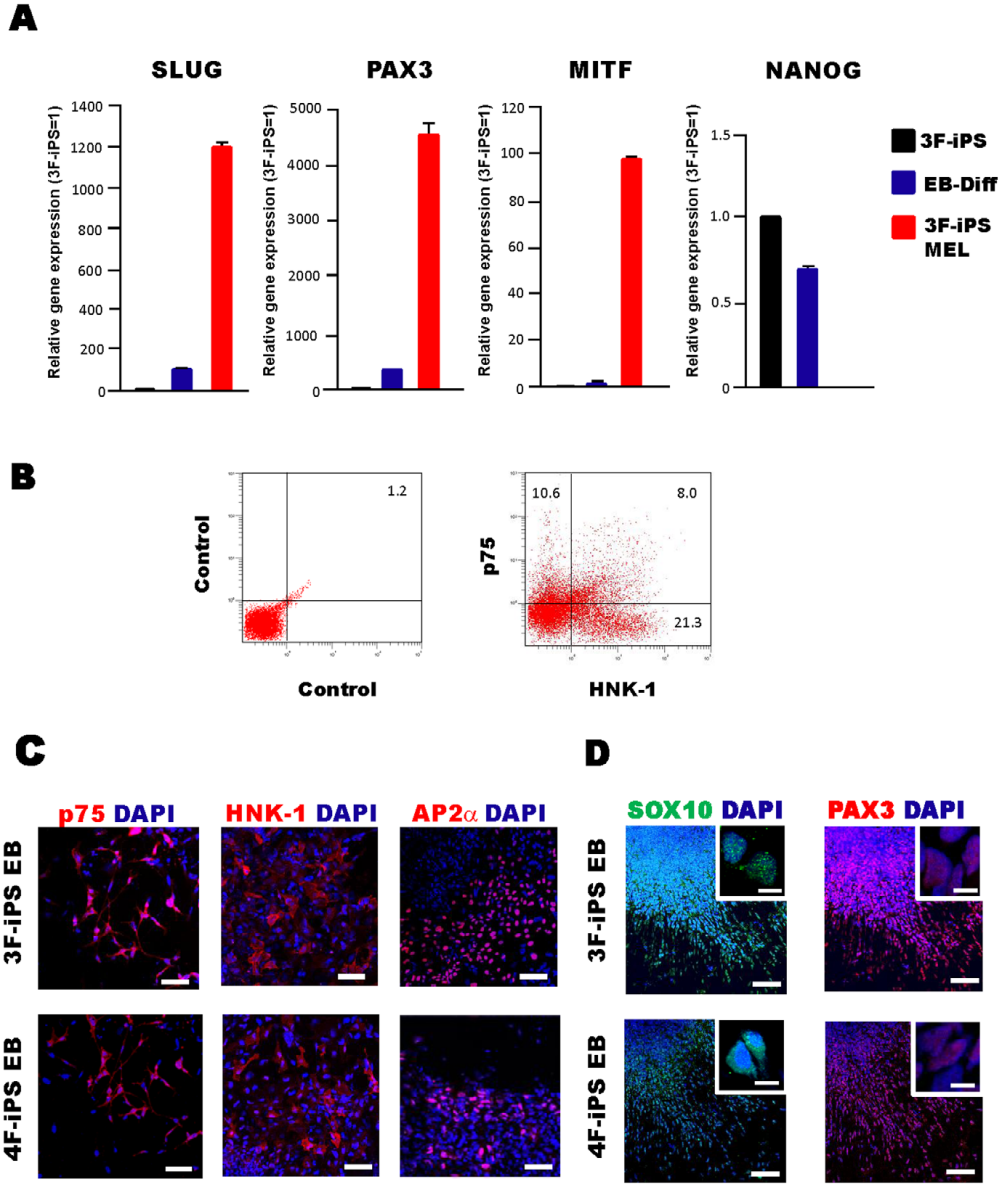
3F- and 4F-iPS cells can be differentiated into NCSC and MELSCs. (A) Gene expression analysis of a neural crest stem cell marker (Slug), a melanocyte stem cell marker (PAX3), a melanocytic marker (MITF), pluripotency marker (NANOG) in 3F-iPS cells, differentiated cells derived from EBs on fibronectin-coated plates in melanocyte differentiation medium at 7 days after differentiation (EB-DIFF), and 3F-iPS derived melanocytes (3F-iPS MEL). Transcript levels were normalized to GAPDH. The graphs show the average of two independent experiments. Error bar indicates mean±S.E.M. (B) Representative flow cytometry results for HNK-1 and p75 staining in 3F-iPS EB-derived cells cultured in melanocyte differentiation medium for one week. Dead cells were excluded using PI staining. (C) Immunocytochemistry revealing cells positive for NCSC markers (p75, HNK-1, and AP2α) in both 3F- and 4F-iPS cells derived EB-differentiated cells 7 days after differentiation. Scale bar, 50 µm. (D) Images show cells positive for neural crest cell marker SOX10, as well as possible melanocyte stem cell marker, PAX3 in both 3F- and 4F-iPS cell-derived EB-differentiated cells 7 days after differentiation. Scale bar, 50 µm and 10 µm (insert images).

Melanocytes are known to be derived from neural crest cells in early development and their numbers are believed to be sustained by melanocyte stem cells in dermal tissues *in vivo*. To identify the cell origin of iPS cell derived-melanocytes in this study, we analyzed expression of the neural crest marker Slug, and one of the putative melanocyte stem cell markers PAX3, melanoblast and melanocyte marker MITF, as well as the pluripotency marker NANOG, using quantitative RT-PCR. The expression of neural crest marker genes was observed in EB-derived cells cultured on fibronectin-coated dishes in melanocyte differentiation medium for 7 days and the gene expression of NANOG was silenced in 3F-iPS MEL (Figure 4A). We also performed flow cytometric and immunocytochemical analyses on EB-derived cells cultured in melanocyte differentiation medium for one week. Flow cytometry showed the presence of neural crest marker p75 and HNK-1 double-positive cells, which are derived from 3F-iPS cell EBs (Figure 4B). Immune-positive cells for the neural crest markers, p75, HNK-1, and AP2α, were observed in migrating cells from EBs one week after differentiation (Figure 4C). The immune-positive cells for a neural crest cell marker, SOX10, [21] and a possible adult melanocyte stem cell marker, PAX3 [22], [23], were also observed in the EBs-derived cells cultured in melanocyte differentiation medium for a week, indicating that the iPS-derived melanocytes may be derived through neural crest cells and melanocyte stem cells as is the case for *in vivo* melanocyte development (Figure 4D).

## Discussion

In this study, we established an *in vitro* method of deriving human melanocytes from iPS cells generated using either four (Oct3/4, SOX2, KLF4, c-MYC) or the three (without c-MYC) Yamanaka factors. Of note, deriving melanocytes from 3F-iPS cells should reduce the risk of tumorigenesis, based on data from a previous study [24]. Developing an *in vitro* human melanocyte differentiation system is important for following the reasons: 1) in addition to understanding human melanocyte biology, melanocytes generated from human iPS cells may be applied to autologous cell transplantation for patients with various pigment cell disorders such as vitiligo; 2) using patient-specific iPS cells, this *in vitro* system may help to reveal the mechanisms of human genetic pigment disorders or even melanoma oncogenesis, particularly through making research on MELSCs and MMSCs more accessible.

During embryonic development, the precursors of melanocytes are non-pigmented melanoblasts derived from the neural crest cells. Developmentally, embryonic melanoblasts differentiate into either mature melanocytes or MELSCs for maintaining the melanocytic system in the adult [21]. Especially in mouse, MELSCs are located in the hair follicle bulge region [7] and appear to be supported by a specialized niche [25], although MELSCs may also be located in other regions including dermis, as shown in humans. In fact, OCT4 positive-human dermal stem cells generated from foreskins lacking hair follicles have been reported to be differentiated into melanocytes [26]. Although candidate MELSC markers, DCT and PAX3, have been reported in mouse [7], [8], [22], it is still difficult to identify MELSCs populations, especially in humans, without more specific markers. In our *in vitro* development system, we observed the appearance of some neural crest cell marker-positive cells one week after EBs differentiation, supporting the notion that iPS-derived melanocytes may be sequentially generated from neural crest cells, mimicking *in vivo* development. In addition to MELSCs [7], human skins contain various stem cell populations such as hair follicle epidermal stem cells [27], hair follicle stem cells [28], bulge neural crest-derived stem cells [29], [30], and skin-derived precursors (SKPs) [31]. It may also be possible to generate such stem cell populations by modifying this culture system starting from iPS cells, eventually leading to better understanding of such human skin progenitor and/or stem cell populations.

Cancer cells including melanoma are suspected to be derived from stem/progenitor cells in which genetic alterations have accumulated [6]. Additionally, cancer stem cells, which have a high tumor initiating ability and cause relapses due to chemotherapy resistance have recently been proposed. Thus, cancer stem cells appear to be an important target for treatment [32]. It was suggested that cancer stem cells may have a similar phenotype as normal tissue stem cells in some cancers. However, the existence and phenotype of MMSCs is still controversial [33], despite various possible markers such as CD20, CD133, CD271, or ABCB5 having been previously suggested [12], [34]. Identification of human MELSCs, or the generation of melanoma cells via melanoma-causing genetic alterations using this in vitro human melanocyte differentiation method from iPS, may lead to further insights into MMSC biology and subsequent development of new treatments targeting MMSCs.

It is important to establish methods to identify good quality human iPS cell clones that are free from tumor development. The safety of the melanocytes generated must still be examined by engraftment in immunodeficient mice. Additionally, based on the percentages of SILV-positive cells, our differentiated cell populations do not appear completely homogeneous. Thus, efforts to increase the purity of differentiated populations, including the removal of immature and/or undifferentiated cells that may be tumorigenic [35], are important to warrant safety prior to clinical cell therapies. In addition, to reduce the risk of tumorigenicity, use of L-MYC instead C-MYC may be an optional choice for the generation of clinical grade iPS cells [36]. In this study, we generated iPS cells using a retrovirus system, and observed imperfect transgene silencing in 3F-iPS as shown in supplemental data; thus, in the future we should develop new methods of generating clinical grade iPS cells without genomic integration of retroviral transgenes, in addition to generating homogeneous iPS clones based on the expression level of the four Yamanaka factors and NANOG.

The technique described herein is especially important for pigment cell disorders, as establishment and expansion of adult melanocytes are both challenging processes. Melanocytes from autologous iPS cells have an obvious advantage over ES cells, due to a negligible chance of immunological rejection, above and beyond surpassing ethical issues. Collectively, it may be important to find technology to select and/or increase iPS cell clones that can generate melanocytes with high yield, as each iPS cell clone may have different differentiation potency. In our culture system, the generation of neural crest cells was observed during the differentiation process. Although developmental models suggest that neural crest cells would also act as the source for melanocytes *in vitro*, we were unable to directly demonstrate this linearity in our differentiation system. We are now optimizing the isolation of intermediate neural crest cells using cell sorting in an attempt to increase the purity and quantity of melanocyte culture. Recently efficient methods to generate neural crest cells from human ES and iPS cells using SMAD inhibition have been reported [37]. Combining our current procedure with SMAD inhibition may ultimately increase the yield of melanocytes through the efficient induction of a transient neural crest cell population. In the future, we predict we will be able to achieve much higher efficiencies of melanocyte derivation by enrichment of either neural crest cells or MELSCs through chemical and/or physical means.

In the present study, we used Wnt3, SCF, ET3, and cAMP inducers (cholera toxin) in our melanocyte differentiation medium. MITF regulates melanocyte lineage by activating pigment-producing genes such as DCT and tyrosinase, and contributes to cell survival by up-regulating anti-apoptotic genes Bcl-2 and BclXL [38]. The MITF promoter is regulated by the transcription factors, PAX3, SOX10, LEF1, and CREB, all of which are downstream signaling molecules of Wnt, ET3, SCF, and cAMP [39]. Therefore, we believe our differentiation conditions bias melanocyte differentiation through a combinatorial activation of MITF. We are currently trying to improve the compositions of our differentiation medium to further increase the yield of melanocytes and reduce the number of components as more simple compositions would be desirable to generate GMP (good manufacturing practice) grade melanocytes for use in future clinical applications.

In summary, we have established *in vitro* differentiation system for human melanocytes from 3F- and 4F-iPS cells. Recently, many patient-specific human iPS cell lines have been established for diseases such as ALS, Familial dysautonomia, Parkinson's disease, and SMA [40]. If differentiated cells from patient-specific iPS cells can faithfully recapitulate the disease phenotype, iPS cell technology will contribute significantly to revealing disease mechanisms and developing drug screening systems for disease. Building on this paradigm, further analysis of the mechanism of melanocyte development using directed differentiation will contribute to not only human melanocyte biology, but also an understanding of various pigment cell disorders leading to the development of new therapeutic strategies.

## Materials and Methods

### Generation of human induced pluripotent stem (iPS) cells and ES cell culture

HDFs (human dermal fibroblasts) from facial dermis of a 36-year-old Caucasian female (Cell Applications Inc, San Diego, CA) were used for the establishment of 4F-iPS cells (OCT3/4, SOX2, KLF4, MYC). 3F-iPS cells (OCT3/4, SOX2, KLF) were generated from primary skin fibroblasts derived from a healthy 16-year-old female volunteer by skin punch biopsy with written informed consent (Keio University School of Medicine). All fibroblasts were maintained in DMEM containing 10% FBS. The induction of human iPS cells was performed as previously described by Takahashi *et al.* [20]. Briefly, fibroblasts lentivirally expressing the mouse receptor for retroviruses (Slc7a1) were seeded at 3×10^5^ cells per 60 mm dish, and infected the next day with retroviruses delivering all four reprogramming factors or three factors without c-MYC. Seven days after transduction, cells were harvested by trypsinization and replated at 5×10^4^−5×10^5^ cells per 100 mm dish on a mitomycin C-treated STO feeder layer (DS Pharma, Osaka, Japan). The next day, the medium was replaced with human iPS medium containing FGF-2 (4 ng/ml, Wako, Osaka, Japan). The medium was changed every other day until colonies were picked. 3F- and 4F-iPS cells, and 201B7 iPS cells [20] were established and characterized following methodology described in the previous study. ES cells (KhES-1) [41] were cultured on the feeder cells in iPS culture medium. Established iPS cells are available upon request to Hideyuki Okano (Keio University).

### Spontaneous *in vitro* differentiation

Embryoid bodies (EBs) were formed from floating culture in EB medium consisting of DMEM/Ham's F12 containing 20% Knockout Serum Replacement (Invitrogen, Carlsbad, CA), 2 mM L-glutamine, 1×10^−4^ M nonessential amino acids, 1×10^−4^ M 2-mercaptoethanol, and 0.5% penicillin and streptomycin in a bacteria dish for 3 weeks. EBs were then transferred to a gelatin-coated dish in the same iPS medium as described above.

### Melanocyte induction from iPS cells

To induce melanocytic differentiation, EBs were derived as described above, however, after three weeks, EBs were plated on a fibronectin-coated dish in DMEM and MCDB 201 based differentiation medium containing 50 ng/ml Wnt3a (R&D Systems, Minneapolis, MN), 50 ng/ml stem cell factor (SCF, R&D Systems), 100 nM Endothelin-3 (ET-3, American Peptide Company, Sunnyvale, CA), 20 pM cholera toxin (Toyobo, Osaka, Japan), 50 nM TPA (12-tetra decanoyl-phorbol 13-acetate, Sigma-Aldrich, St Louis, MO), 4 ng/ml FGF-2 (WAKO), 100 µM L-ascorbic acid (Sigma), 50 nM dexamethasone (Sigma), 1 mg/ml linoleic acid-bovine serum albumin (Sigma), and 1X insulin-transferrin-selenium (Sigma) following the method described by Fang *et al.* [14]. Before attaining confluence, cells were dissociated using TrypLE Select (Invitrogen) and replated onto a fibronectin (BD Biosciences, San Jose, CA)-coated dish in differentiation medium until the appearance of pigmented cells and continued to be cultured for few months.

### Human melanocyte culture cells

Cultured normal human foreskin-derived epidermal melanocyte (NHEM) cells were obtained from Kurabo (Osaka, Japan). Melanocytes were maintained in melanocyte complete 254 culture medium (Kurabo) supplemented with 0.5% fetal bovine serum, 5 µg/ml insulin, 0.5 µM hydrocortisone, 5 µg/ml transferring, 3 µg/ml heparin, 3 ng/ml human FGF-2, 10 ng/ml phorbol-12-myristate-13-acetate, and 0.2% bovine pituitary extract.

### Teratoma formation

For iPS injection into the testis of 8-week-old NOD/SCID mice (OYC International, Tokyo, Japan), a 1 cm longitudinal incision in the abdominal wall was made, and the testis was exteriorized. One million iPS cells were inoculated into the testis parenchyma with a Hamilton pipette, and the wound was sutured. Eight weeks after injection, tumors were dissected and fixed with PBS containing 4% paraformaldehyde (PFA). Paraffin-embedded tissues were sectioned and stained with hematoxylin and eosin.

### RT-PCR

Total RNA was purified with Trizol reagent (Invitrogen) and PureLink RNA Mini Kit (Invitrogen) after DNAase I treatment to remove genomic DNA. cDNA synthesis was performed using High Capacity RNA-to-cDNA Master Mix (Applied Biosystems, Forster City, CA) according to the manufacturer's instructions. The primers were designed as follows: c-Kit forward primer, 5′-GTTCTGCTCCTACTGCTTCGC-3′; reverse primer, 5′-TAACAGCCTAATCTCGTCGCC-3′, MITF forward primer, 5′-TGCCCAGGCATGAACACAC-3′; reverse primer, 5′-TGGGAAAAATACACGCTGTGAG-3′; MITF-M forward primer, 5′-ACCTTCTCTTTGCCAGTCCATCT-3′; reverse primer,5′-GGACATGCAAGCTCAGGACT-3, PAX3 forward primer, 5′-AGCCGCATCCTGAGAAGTAA-3′; reverse primer, 5′-CTTCATCTGATTGGGGTGCT-3′, Slug forward primer, 5′-CATACAGCCCCATCACTGTG-3′; reverse primer, 5′-CTTGGAGGAGGTGTCAGATG-3′; GAPDH, forward primer, 5′-TGAACGGGAAGCTCACTGGG-3′; reverse primer,TCCACCACCCTGTTGCTGTA-3′.

For the detection of gene expression of OCT3/4, SOC2, KLF4, c-MYC, NANOG, and REX1, primer sets published in Takahashi *et al.* [20] were used. The detection of transgene expression, we used sequence specific primer sets for these genes. Quantitative PCR was performed using Thunderbird syber qPCR Mix (Toyobo). Transcript levels were determined using the ABI PRISM Sequence detection System 7900HT (Applied BioSystems). Gene expression was normalized to GAPDH as an internal standard.

### Bisulfite Sequencing

Genomic DNA was purified from each set of cells using a Wizard SV Genomic DNA Purification Kit (Promega, Tokyo, Japan). Bisulfite conversion was performed with 500 ng of genomic DNA using the reagents provided in Qiagen's EpiTect Bisulfate kit (Qiagen, Tokyo, Japan). The promoter region of human OCT3/4 (−2344 to −2126) was amplified using primers following the study by Freberg *et al.* [42]; forward primer, 5′-ATTTGTTTTTTGGGTAGTTAAAGGT-3′; reverse primer, 5′-CCAACTATCTTCATCTTAATAACATCC-3′. The PCR products were subcloned into pGEM-T Easy (Promega) and sequenced. Twelve clones of each sample were verified by sequencing with the T7 universal primer.

### DNA Microarray

Approximately 10^6^ cells were used for total RNA extraction using the PureLink RNA Mini Kit (Invitrogen), according to the manufacturer's instructions. RNA quality was verified with the Bioanalyser System (Agilent Technologies, Paolo Alto, CA), using RNA Nano Chips. 1 µg of RNA was processed for hybridization on the Whole Human Genome Microarray4×44k (Agilent). Processing was done according to the recommendations of the manufacturer. All genomic and transcriptomic analysis was carried using GeneSpring software 7.3.1. (Agilent). Signal intensities less than 0.01 were set to 0.01, then each chip was normalized to the 50th percentile of the measurements taken from that chip.

### Flow cytometry

Cells were analyzed using an EPICS XL instrument (Beckman-Coulter). Prior to flow cytometry, cells were dissociated with Accumax (Innovative Cell Technologies, San Diego, CA) for 15 min at room temperature and then filtrated with 40 µm cell strainers. CD57 (HNK-1) FITC-conjugated antibody (Beckman-Coulter), CD271 (p75) PE- conjugated antibody (Biolegend, San Diego, CA), Mouse IgG-PE isotype antibody, and Mouse IgM-FITC isotype antibody were used for immunolabeling. Propidium Iodide (PI) staining excluded dead cells.

### Immunocytochemical analysis and alkaline phosphatase staining

For immunocytochemistry, cells were fixed with phosphate buffered saline (PBS) containing 4% PFA for 20 min at room temperature. Then, cells were subjected to immunofluorescence staining using the following primary antibodies: α-fetoprotein (AFP) (1∶500, DAKO, Kyoto, Japan), α-smooth muscle actin (SMA) (1∶100, Sigma), AP2α (1∶50, Cell Signaling Technology, Beverly, MA), β-III-tubulin (1∶1000, Sigma), microphthalmia-associated transcription factor (MITF) (1∶50, Thermo Scientific, Rockford, IL), HNK-1 (1∶200, Millipore, Billerica, MA), NANOG (1∶1000, Reprocell, Tokyo, Japan), OCT3/4 (1∶200, BD Bioscience, San Diego, CA), p75 (1∶100, Advanced Targeting Systems), PAX3 (1∶100, R&D Systems), S100 (1∶500, DAKO), silver protein (SILV) (1∶50, DAKO), SOX10 (1∶100, Abcam), SSEA-4 (1∶200, Millipore), TRA-1-60 (1∶200, Millipore), TRA-1-81 (1∶200, Millipore), tyrosinase (TYR) (1∶100, Santa Cruz Biothechnology, Inc., Santa Cruz, CA), tyrosinase-related protein-1 (TYRP1) (1∶20, COVANCE, Princeton, NJ). After PBS washes, antibody binding was visualized using either Alexa Fluor 488 or 546-conjugated secondary antibodies (Invitrogen), and the nuclei were stained with DAPI (4′,6-diamino-2-phenylindole). Images were obtained using a Zeiss LSM-510 confocal microscope (Zeiss, Tokyo, Japan). Alkaline phosphatase staining was performed using the Leukocyte Alkaline Phosphatase kit (Sigma).

### Electron microscopy

Cells were fixed with 2.5% glutaraldehyde and 4% PFA in 0.1 M cacodylate buffer (pH 7.4) and post-fixed with 1% OsO_4_. Images were obtained using an electron microscope (Japanese Electronic Optical Laboratories, JEOL-1230).

## Supporting Information

Figure S1 Quantitative PCR for expression of retroviral transgenes in human iPS cells.
(TIF)

Figure S2 Analysis of differentiated cells in teratoma.
(TIF)
