# Correction: Generation of Human Melanocytes from Induced Pluripotent Stem Cells

**DOI:** 10.1371/journal.pone.0337375

**Published:** 2025-11-24

**Authors:** Shigeki Ohta, Yoichi Imaizumi, Yohei Okada, Wado Akamatsu, Reiko Kuwahara, Manabu Ohyama, Masayuki Amagai, Yumi Matsuzaki, Shinya Yamanaka, Hideyuki Okano, Yutaka Kawakami

The Fig 4C 3F-iPS EB panel, and the Fig 4D 3F-iPS EB SOX10 and PAX3 panels are incorrect. The authors provided an updated Fig 4 presenting the correct panels and the triplicate image data underlying the Fig 4C and Fig 4D results [S1-S2 File]. The enlarged images in the original Fig 4D 4F-iPS EB SOX10 DAPI and 4F-iPS EB PAX3 DAPI were replaced with clearer images from the same samples. The authors state that the underlying data for Figs 1-3, and Figs 4A-B remain available upon request.

**Fig 4:**
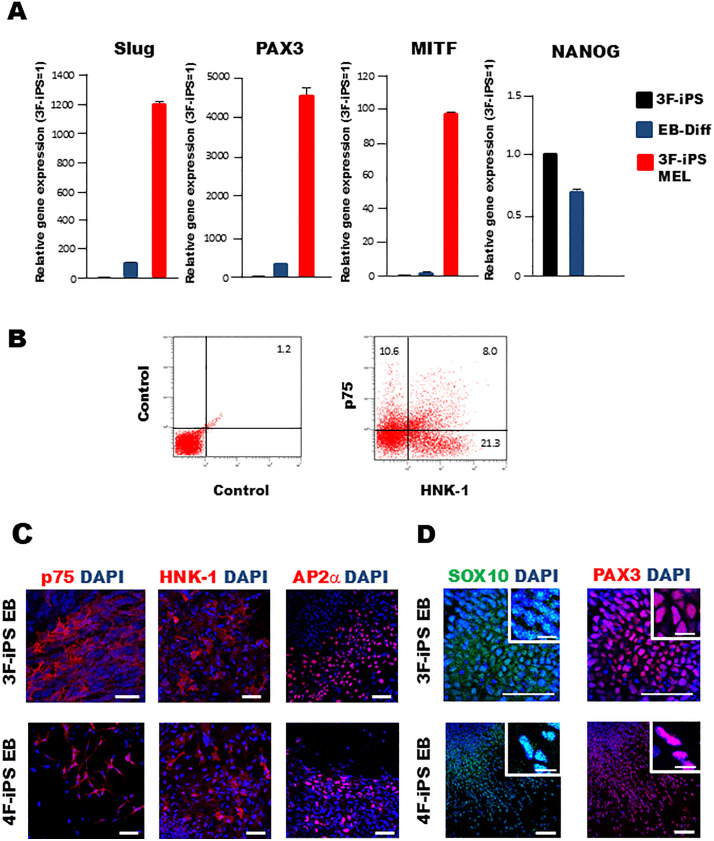
3F- and 4F-iPS cells can be differentiated into NCSC and MELSCs. (A) Gene expression analysis of a neural crest stem cell marker (Slug), a melanocyte stem cell marker (PAX3), a melanocytic marker (MITF), pluripotency marker (NANOG) in 3F-iPS cells, differentiated cells derived from EBs on fibronectin-coated plates in melanocyte differentiation medium at 7 days after differentiation (EB-Diff), and 3F-iPS derived melanocytes (3F-iPS MEL). Transcript levels were normalized to GAPDH. The graphs show the average of two independent experiments. Error bar indicates mean±S.E.M. (B) Representative flow cytometry results for HNK-1 and p75 staining in 3F-iPS EB-derived cells cultured in melanocyte differentiation medium for one week. Dead cells were excluded using PI staining. (C) Immunocytochemistry revealing cells positive for NCSC markers (p75, HNK-1, and AP2α) in both 3F- and 4F-iPS cells derived EB-differentiated cells 7 days after differentiation. Scale bar, 50 µm. (D) Images show cells positive for neural crest cell marker SOX10, as well as possible melanocyte stem cell marker, PAX3 in both 3F- and 4F-iPS cell-derived EB-differentiated cells 7 days after differentiation. Scale bar, 50 µm and 10 µm (insert images).

## Supporting information

S1 File Triplicate image data underlying the Fig 4C results. (ZIP)

S2 File Triplicate image data underlying the Fig 4D results. (ZIP)
