# Supplementary figures and images for: Generation of Human Melanocytes from Induced Pluripotent Stem Cells
Source: PLoS One. 2011 Jan 13;6(1):e16182. doi: 10.1371/journal.pone.0016182 (PMC3020956; doi:10.1371/journal.pone.0016182)

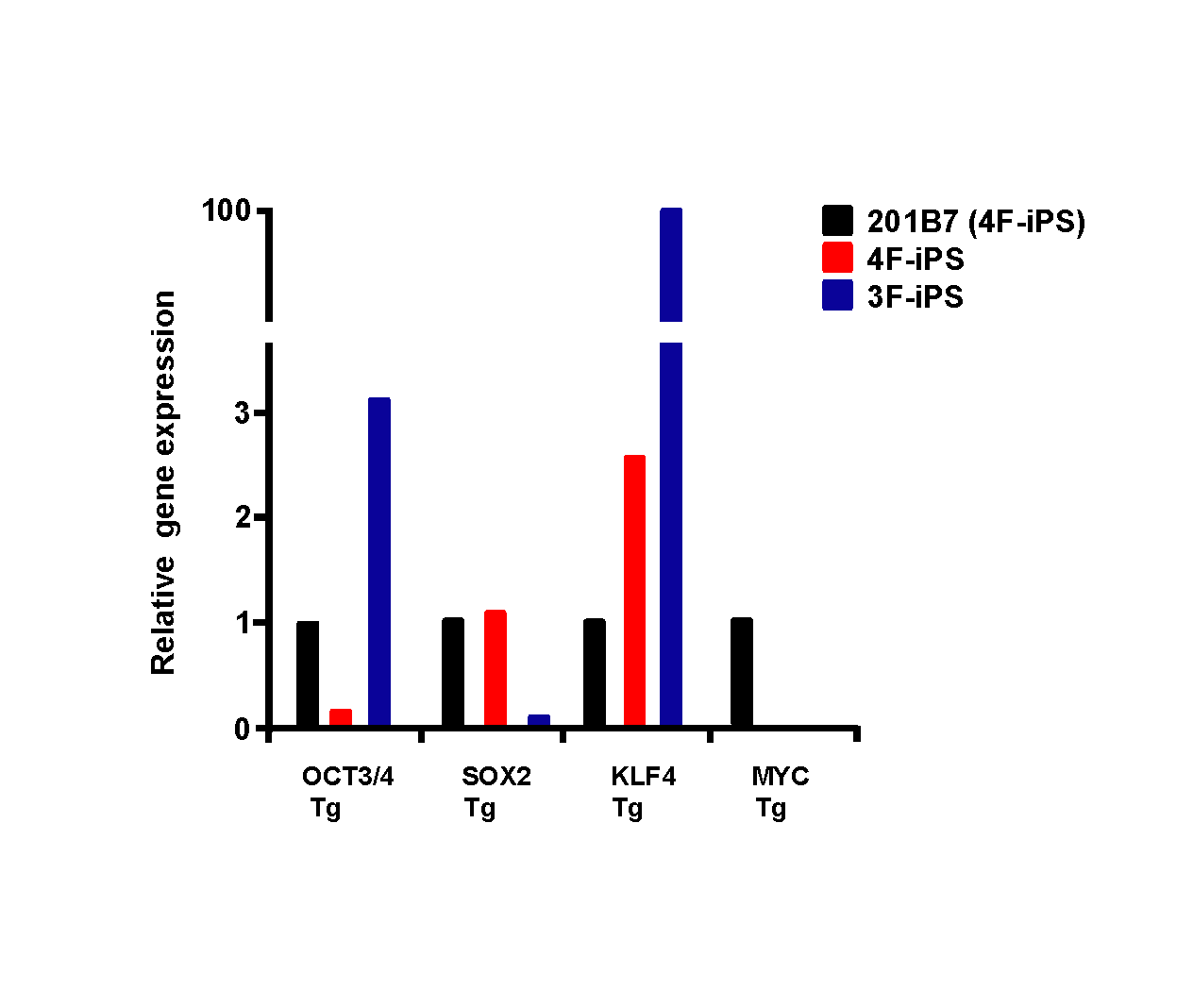

Supplement: Figure S1 — Quantitative PCR for expression of retroviral transgenes in human iPS cells. (TIF) [file pone.0016182.s001.tif]

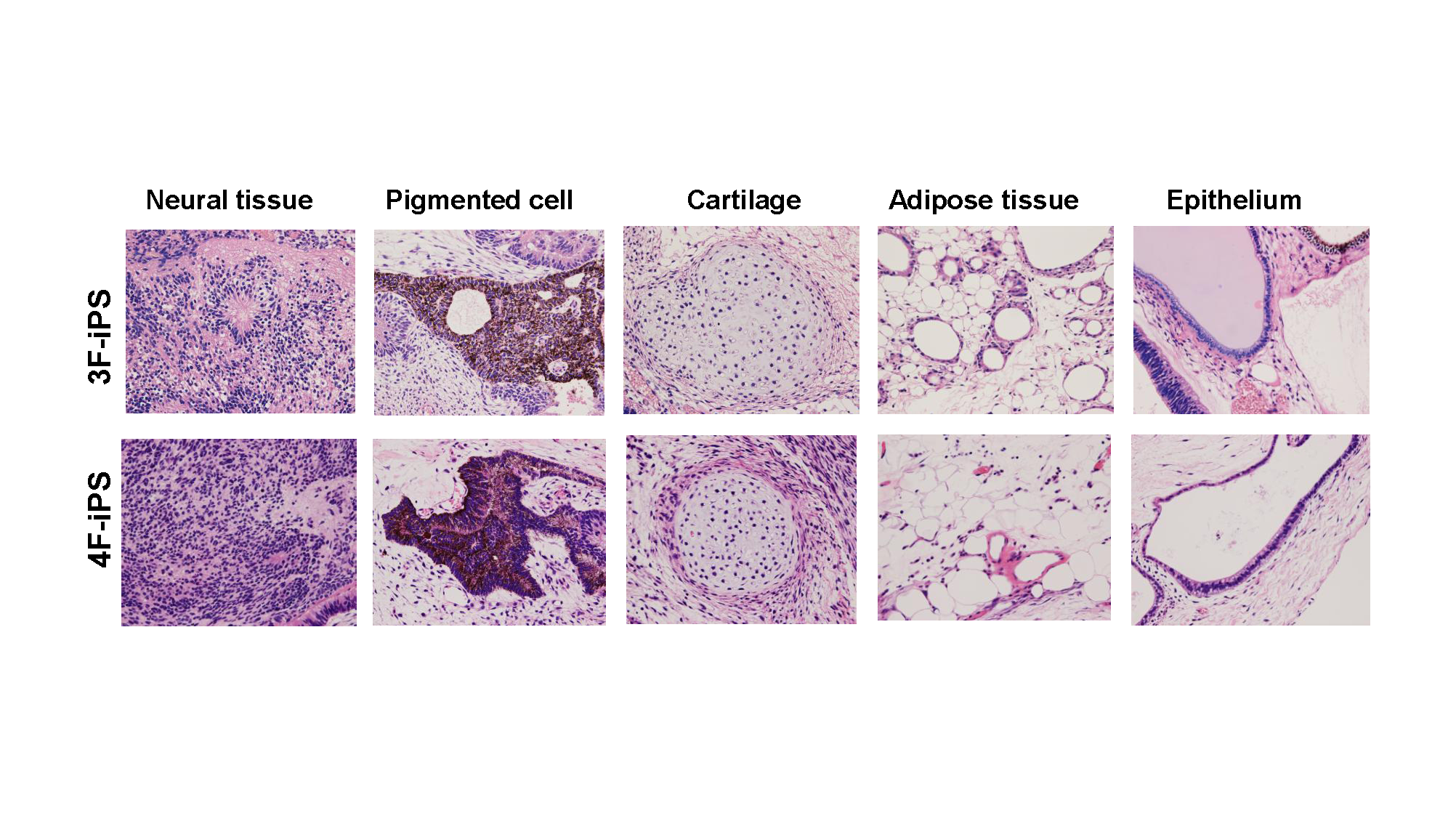

Supplement: Figure S2 — Analysis of differentiated cells in teratoma. (TIF) [file pone.0016182.s002.tif]
