# Supplementary figures and images for: Correction: Generation of Human Melanocytes from Induced Pluripotent Stem Cells
Source: PLoS One. 2025 Nov 24;20(11):e0337375. doi: 10.1371/journal.pone.0337375 (PMC12643287; doi:10.1371/journal.pone.0337375)

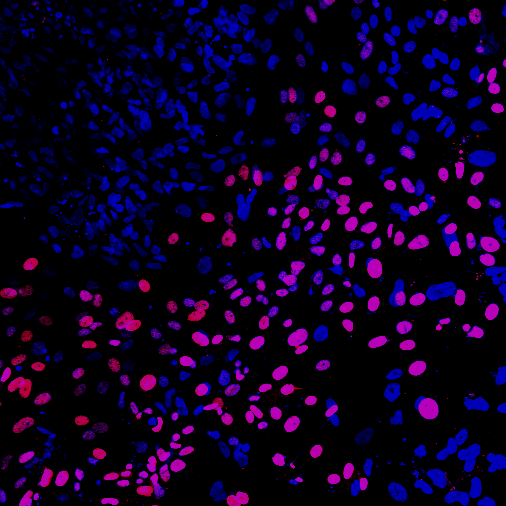

Supplement: S1 File — (ZIP) [file pone.0337375.s001.zip › S1 File/3F-iPS AP2α/3F-iPS-AP2a-1.tif]

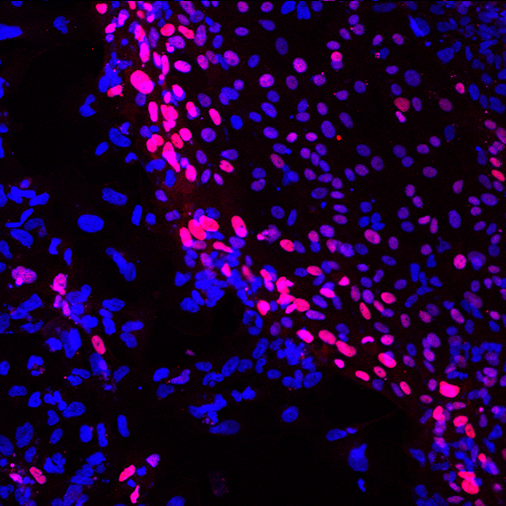

Supplement: S1 File — (ZIP) [file pone.0337375.s001.zip › S1 File/3F-iPS AP2α/3F-iPS-AP2a-2.tif]

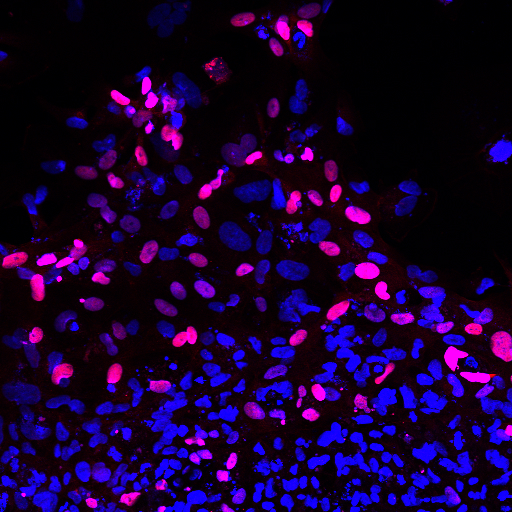

Supplement: S1 File — (ZIP) [file pone.0337375.s001.zip › S1 File/3F-iPS AP2α/3F-iPS-AP2a-3.tif]

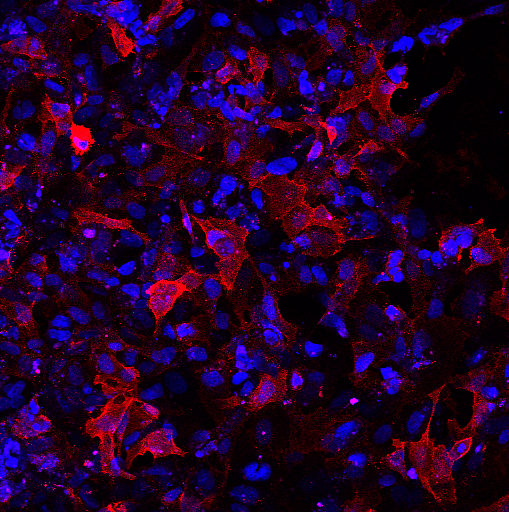

Supplement: S1 File — (ZIP) [file pone.0337375.s001.zip › S1 File/3F-iPS HNK1/3F-iPS-HNK-1-1.tif]

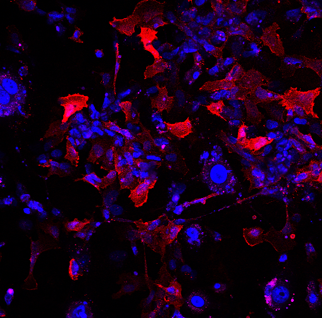

Supplement: S1 File — (ZIP) [file pone.0337375.s001.zip › S1 File/3F-iPS HNK1/3F-iPS-HNK-1-2.tif]

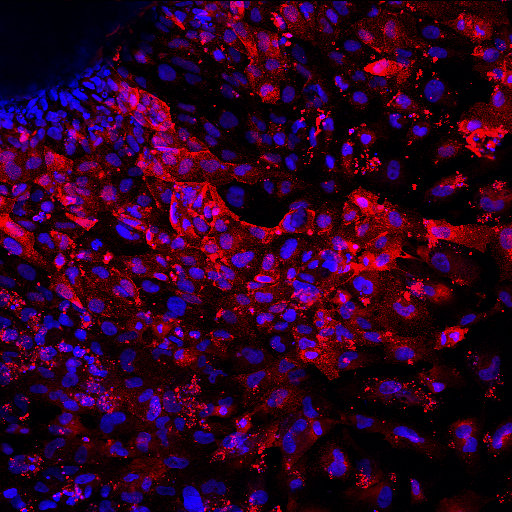

Supplement: S1 File — (ZIP) [file pone.0337375.s001.zip › S1 File/3F-iPS HNK1/3F-iPS-HNK-1-3.tif]

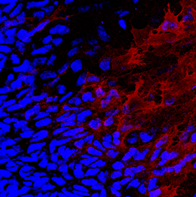

Supplement: S1 File — (ZIP) [file pone.0337375.s001.zip › S1 File/3F-iPS p75/3F-iPSp75(3).jpg]

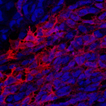

Supplement: S1 File — (ZIP) [file pone.0337375.s001.zip › S1 File/3F-iPS p75/3F-p75(1).jpg]

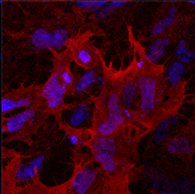

Supplement: S1 File — (ZIP) [file pone.0337375.s001.zip › S1 File/3F-iPS p75/3F-p75(2).jpg]

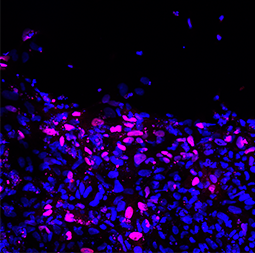

Supplement: S1 File — (ZIP) [file pone.0337375.s001.zip › S1 File/4F-iPS AP2α/4F-iPS-AP2a-1.tif]

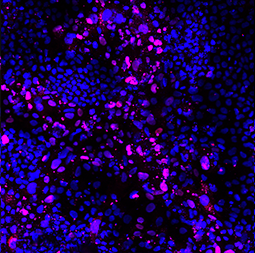

Supplement: S1 File — (ZIP) [file pone.0337375.s001.zip › S1 File/4F-iPS AP2α/4F-iPS-AP2a-2.tif]

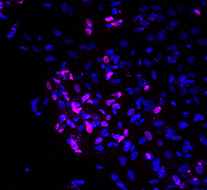

Supplement: S1 File — (ZIP) [file pone.0337375.s001.zip › S1 File/4F-iPS AP2α/4F-iPS-AP2a-3.tif]

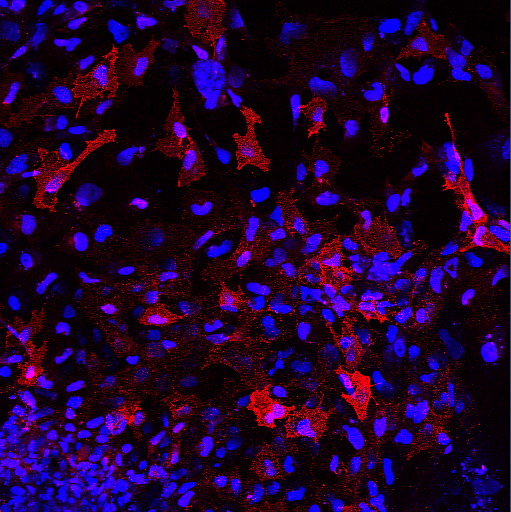

Supplement: S1 File — (ZIP) [file pone.0337375.s001.zip › S1 File/4f-iPS HNK1/4F-iPS-HNK-1-1.tif]

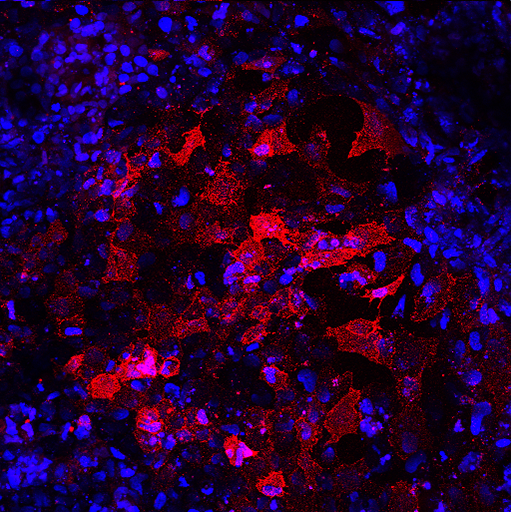

Supplement: S1 File — (ZIP) [file pone.0337375.s001.zip › S1 File/4f-iPS HNK1/4F-iPS-HNK-1-2.tif]

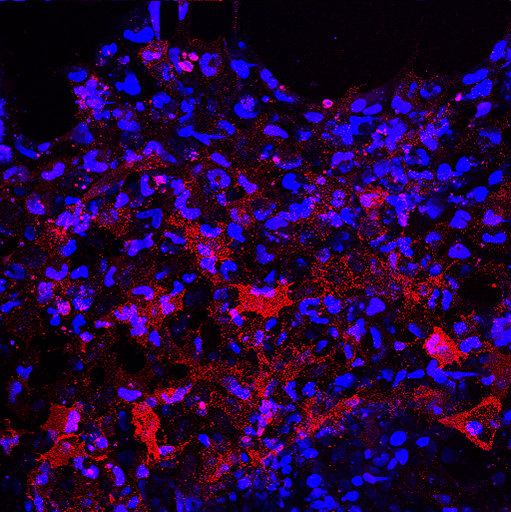

Supplement: S1 File — (ZIP) [file pone.0337375.s001.zip › S1 File/4f-iPS HNK1/4F-iPS-HNK-1-3.tif]

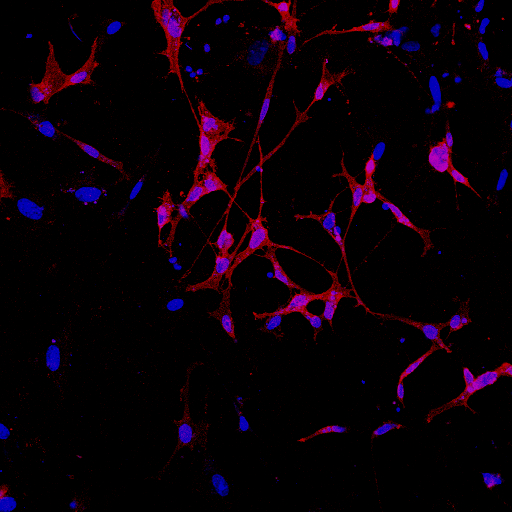

Supplement: S1 File — (ZIP) [file pone.0337375.s001.zip › S1 File/4F-iPS p75/4F-iPS-p75-1.tif]

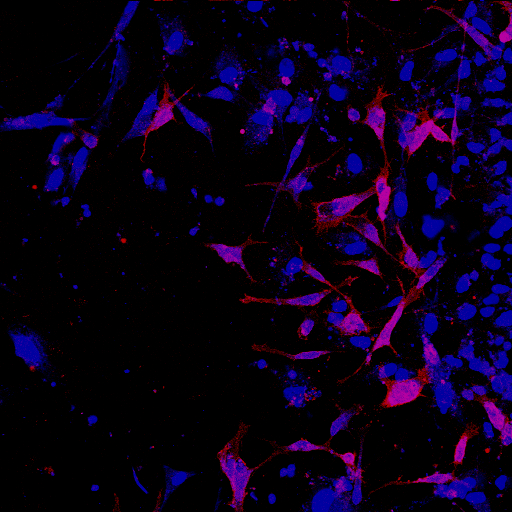

Supplement: S1 File — (ZIP) [file pone.0337375.s001.zip › S1 File/4F-iPS p75/4F-iPS-p75-2.tif]

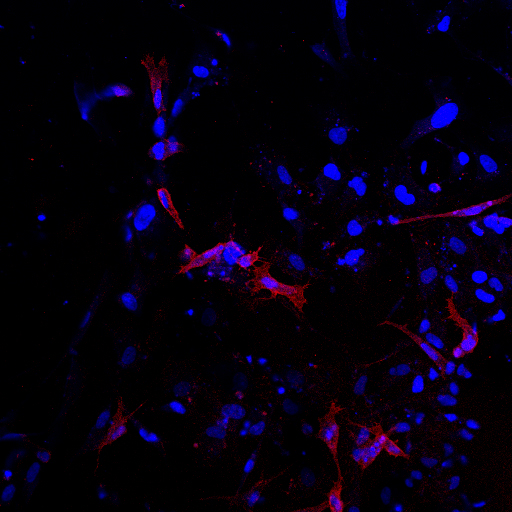

Supplement: S1 File — (ZIP) [file pone.0337375.s001.zip › S1 File/4F-iPS p75/4F-iPS-p75-3.tif]

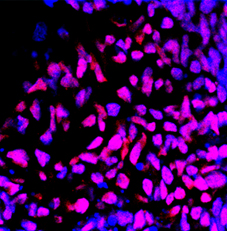

Supplement: S2 File — (ZIP) [file pone.0337375.s002.zip › S2 File/3F-iPS PAX3/3F-iPS (3-1).jpg]

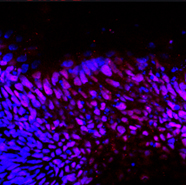

Supplement: S2 File — (ZIP) [file pone.0337375.s002.zip › S2 File/3F-iPS PAX3/3F-iPS PAX3 (2-1).jpg]

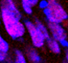

Supplement: S2 File — (ZIP) [file pone.0337375.s002.zip › S2 File/3F-iPS PAX3/3F-iPS PAX3 (2-2).jpg]

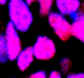

Supplement: S2 File — (ZIP) [file pone.0337375.s002.zip › S2 File/3F-iPS PAX3/3F-iPS PAX3 (3-2).jpg]

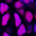

Supplement: S2 File — (ZIP) [file pone.0337375.s002.zip › S2 File/3F-iPS PAX3/3F-iPS PAX3(1-2).jpg]

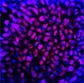

Supplement: S2 File — (ZIP) [file pone.0337375.s002.zip › S2 File/3F-iPS PAX3/3F-iPS PAX3-1(1-1).jpg]

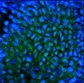

Supplement: S2 File — (ZIP) [file pone.0337375.s002.zip › S2 File/3F-iPS SOX10/3F-iPS SOX10 (1-1).jpg]

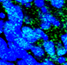

Supplement: S2 File — (ZIP) [file pone.0337375.s002.zip › S2 File/3F-iPS SOX10/3F-iPS SOX10 (2-2).jpg]

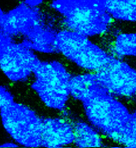

Supplement: S2 File — (ZIP) [file pone.0337375.s002.zip › S2 File/3F-iPS SOX10/3F-iPS SOX10(1-2).jpg]

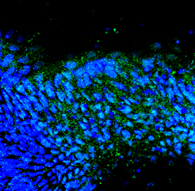

Supplement: S2 File — (ZIP) [file pone.0337375.s002.zip › S2 File/3F-iPS SOX10/3F-iPS SOX10(2-1).jpg]

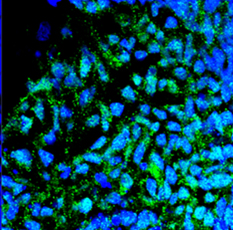

Supplement: S2 File — (ZIP) [file pone.0337375.s002.zip › S2 File/3F-iPS SOX10/3F-iPS SOX10(3-1).jpg]

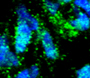

Supplement: S2 File — (ZIP) [file pone.0337375.s002.zip › S2 File/3F-iPS SOX10/3F-iPS SOX10(3-2).jpg]

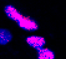

Supplement: S2 File — (ZIP) [file pone.0337375.s002.zip › S2 File/4F-iPS PAX3/4-iPS-PAX3-1B.tif]

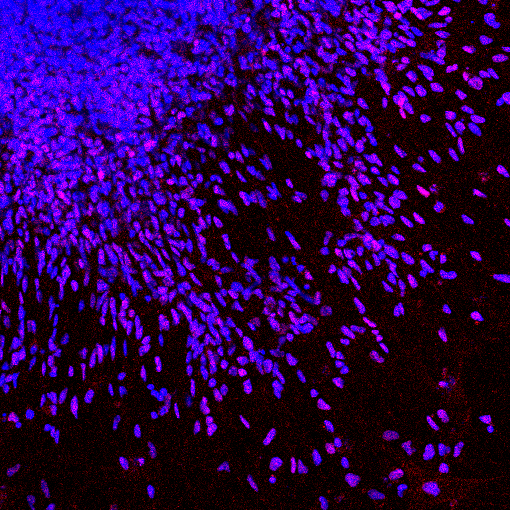

Supplement: S2 File — (ZIP) [file pone.0337375.s002.zip › S2 File/4F-iPS PAX3/4f-iPS-PAX3-1A.tif]

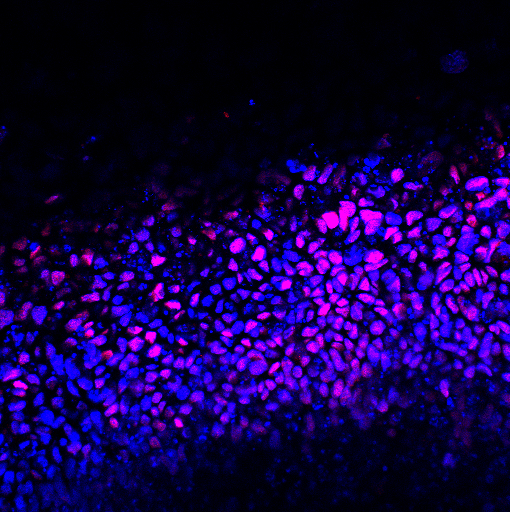

Supplement: S2 File — (ZIP) [file pone.0337375.s002.zip › S2 File/4F-iPS PAX3/4f-iPS-PAX3-2A.tif]

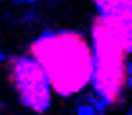

Supplement: S2 File — (ZIP) [file pone.0337375.s002.zip › S2 File/4F-iPS PAX3/4f-iPS-PAX3-2B.tif]

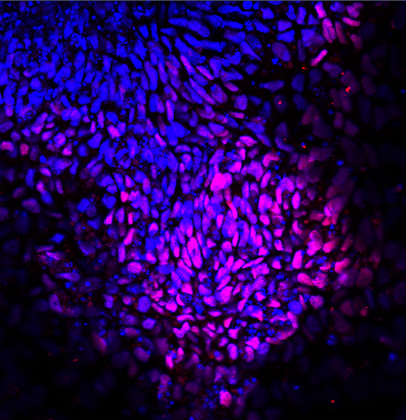

Supplement: S2 File — (ZIP) [file pone.0337375.s002.zip › S2 File/4F-iPS PAX3/4F-iPS-PAX3-3A.tif]

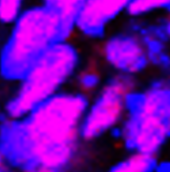

Supplement: S2 File — (ZIP) [file pone.0337375.s002.zip › S2 File/4F-iPS PAX3/4F-iPS-PAX3-3B.tif]

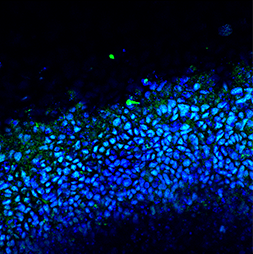

Supplement: S2 File — (ZIP) [file pone.0337375.s002.zip › S2 File/4F-iPS SOX10/4F-iPS-SDOX10-2A.tif]

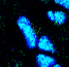

Supplement: S2 File — (ZIP) [file pone.0337375.s002.zip › S2 File/4F-iPS SOX10/4F-iPS-SOX10-1B.tif]

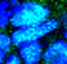

Supplement: S2 File — (ZIP) [file pone.0337375.s002.zip › S2 File/4F-iPS SOX10/4F-iPS-SOX10-2B.tif]

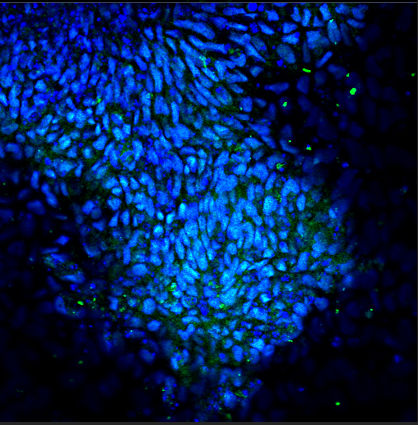

Supplement: S2 File — (ZIP) [file pone.0337375.s002.zip › S2 File/4F-iPS SOX10/4F-iPS-SOX10-3A.tif]

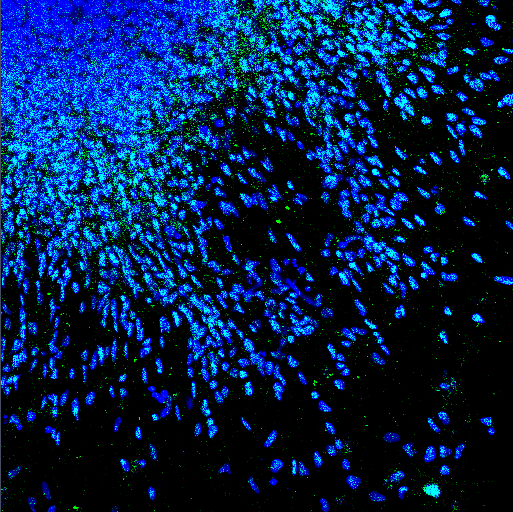

Supplement: S2 File — (ZIP) [file pone.0337375.s002.zip › S2 File/4F-iPS SOX10/4F-SOX10-1A.jpg]

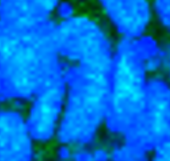

Supplement: S2 File — (ZIP) [file pone.0337375.s002.zip › S2 File/4F-iPS SOX10/4F-SOX10-3B.tif]
